# Reputation-Based Spectrum Sensing Strategy Selection in Cognitive Radio Ad Hoc Networks

**DOI:** 10.3390/s18124377

**Published:** 2018-12-11

**Authors:** Zhiguo Sun, Zhenyu Xu, Zengmao Chen, Xiaoyan Ning, Lili Guo

**Affiliations:** College of Information and Communication Engineering, Harbin Engineering University, Harbin 150001, China; sunzhiguo@hrbeu.edu.cn (Z.S.); xuzhenyuaichiyu@hrbeu.edu.cn (Z.X.); chenzengmao@hrbeu.edu.cn (Z.C.); guolili@hrbeu.edu.cn (L.G.)

**Keywords:** CRAHNs, collaborative spectrum sensing, SSDF, spectrum sensing strategy selection

## Abstract

Spectrum sensing plays an essential role in the detection of unused spectrum whole in cognitive radio networks, including cooperative spectrum sensing (CSS) and independent spectrum sensing. In cognitive radio ad hoc networks (CRAHNs), CSS enhances the sensing performance of cognitive nodes by exploring the spectrum partial homogeneity and fully utilizing the knowledge of neighboring nodes, e.g., sensing results and topological information. However, CSS may also open a door for malicious nodes, i.e., spectrum sensing data falsification (SSDF) attackers, which report fake sensing results to deteriorate the performance of CSS. Generally, the performance of CSS has an inverse relationship with the fraction of SSDF attackers. On the contrary, independent spectrum sensing is robust to SSDF attacks. Therefore, it is desirable to choose a proper sensing strategy between independent sensing and collaborative sensing for CRAHNs coexisting with various fractions of SSDF attackers. In this paper, a novel algorithm called Spectrum Sensing Strategy Selection (4S) is proposed to select better sensing strategies either in a collaborative or in an independent manner. To derive the maximum a posteriori estimation of nodes’ spectrum status, we investigated the graph cut-based CSS method, through which the topological information cost function and the sensing results cost function were constructed. Moreover, the reputation value was applied to evaluate the performance of CSS and independent sensing. The reputation threshold was theoretically analyzed to minimize the probability of choosing the sensing manner with worse performance. Simulations were carried out to verify the viability and the efficiency of the proposed algorithm.

## 1. Introduction

With the rapid development of radio communication, radio systems tend to provide higher speed, have denser deployment, and occupy wider bandwidth, which can cause the radio spectrum to become overcrowded, i.e., spectrum scarcity. However, many studies have shown that under the current radio communication strategy, spectrum resources have not been fully utilized [1]. This provides a possibility to resolve the contradiction between limited spectrum and the increasing requirement of radio communication bandwidth. To address the problem of spectrum scarcity and improve spectrum utilization, cognitive radio has been proposed and widely studied as a promising technology [2].

In cognitive radio networks, the primary users (PUs) are licensed to occupy the authorized frequency band. To avoid interfering with the PU, the secondary users (SUs) can opportunistically access the frequency band, which is not fully utilized by the PUs, i.e., the spectrum white holes [3]. When PUs are active, spectrum resources are not available for SUs; SUs can access the spectrum opportunistically through spectrum sensing when PUs are mute. Therefore, spectrum sensing is essential for cognitive radio to exploit spectrum holes. To avoid interfering with the PU, the spectrum sensing algorithm should be able to detect white holes efficiently and effectively.

Due to the spacious coverage of ad hoc networks, each SU may receive varying PU signal powers and thus undergo diverse spectral status in cognitive radio ad hoc networks (CRAHNs). However, neighboring SUs are spatially close and prefer to experience identical spectral status. This characteristic of ad hoc networks is called spectrum partial homogeneity [4]. Cooperative spectrum sensing (CSS) takes full advantage of this prior information to improve the performance of malfunctioning SUs with poor wireless propagation characteristics, such as those nodes experiencing deep shading. It is unlikely that all SUs undergo severe fading. Therefore, it is feasible for SUs to exploit sensing performance gain from adjacent nodes through cooperation. In the first step of collaborative sensing, each SU reports the local independent spectrum sensing decision to the data fusion center through control channels. Then, the data fusion center generalizes the cooperation of the local decision according to given rules, such as Majority criteria, Or criteria, And criteria, Bayes criterion, and so on [5]. The decision results are broadcast back to each SU node via the control channels.

On one hand, introducing cooperation during sensing provides CRAHNs with enormous performance improvement; on the other hand, it also opens the door to spectrum sensing data falsification (SSDF) attackers, which aim to undermine the sensing performance of CRAHNs. Moreover, ad hoc networks are more likely to sustain SSDF attack due to the openness and dynamics of CRAHNs. Hence, it is crucial to design robust collaborative sensing algorithms to suppress SSDF attackers. The issue of how to eliminate the interference of SSDF attackers in cognitive radio has attracted the attention of many researchers [6,7,8,9,10]. Reputation value theory (RVT)-based algorithms against SSDF attacks are commonly applied in cognitive radio networks to detect and remove malicious nodes [11,12]. However, existing RVT algorithms cannot detect all malicious SUs and even regard honest SUs as malicious nodes by mistake. Residual malicious nodes and mis-detected honest nodes will inevitably degrade the sensing performance. In Reference [13], the maximum likelihood estimation method is combined with reputation theory to eliminate SSDF attackers. The performance of independent sensing is compared with the performance of CSS under SSDF attack in Reference [14]. Unfortunately, the independent sensing results, which perform better when the fraction of SSDF attackers is high, are not used in the proposed algorithm [14]. Different from previous researches, this paper mainly concentrates on the problem of choosing the sensing strategy between either independent spectrum sensing or CSS from the view of honest Sus, rather than how to reject malicious nodes from the view of the data fusion center.

The contributions of this article are as follows:

- The theoretical deduction of the maximum posterior probability estimation based on image segmentation in CRAHNs is introduced.
- This paper proposes a spectrum sensing strategy selection algorithm based on the reputation value theory. Moreover, the theoretical derivation and simulation verification of the optimal threshold value are completed.

The arrangement of this paper is as follows: Section 2 establishes the system model of a cognitive radio network. Section 3 introduces the spectrum sensing methods in cognitive radio networks, and Section 4 proposes the selection strategy of spectrum sensing methods. Section 5 investigates the optimal threshold for the spectrum sensing strategy selection algorithm. Simulation results are given, and conclusions are drawn in Section 6. Finally, Section 7 discusses the potential future works that may be needed.

## 2. System Model

**Figure 1:**
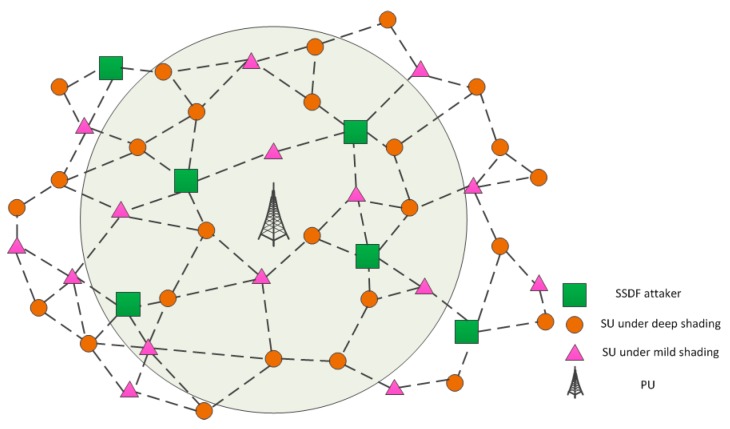
This figure shows a centralized cognitive radio ad hoc network (CRAHN) contaminated by some spectrum sensing data falsification (SSDF) attackers. The gray circle around the primary user (PU) indicates the coverage of PU. Inside the circle, the secondary user (SU) should not access the spectrum while SUs outside the circle have this opportunities.

This paper considers a centralized CRAHN model, consisting of a PU, a fusion center (FC), and N SUs with N−M Honest Secondary Users (HSUs) and M malicious secondary users (MSUs). As shown in Figure 1, parts of the secondary users are affected by severe fading and uncertain noise, resulting in false sensing decisions. Thereby, we exploit the prior information of the spectral state consistency of CRAHNs to improve the performance of these fading nodes via cooperation. Although the cognitive nodes of different location may experience different spectrum status, topologically adjacent nodes tend to be in the same spectrum occupancy status. In other words, CRAHNs are composed of several ‘same spectrum status’ patches. Therefore, malfunctioning SUs under deep fading can cooperate with neighboring nodes to rectify the local erroneous spectrum sensing results.

In the first step of CSS, the SUs carry out independent spectrum sensing, and then send the local sensing decisions to the FC through the ideal control channels. In ad hoc networks, the topology information is relatively easy to acquire. Based on the accepted local sensing results and known topology information, the FC makes a final decision, and then broadcasts the sensing decisions to all SUs.

At each cognitive node, independent spectrum sensing can be considered as a binary hypothesis testing problem defined as:

$$\{\begin{array}{cc} H_{1}: & the\;spectrum\;is\;occupied\\ H_{0}: & the\;spectrum\;is\;idle \end{array}$$

The power of the PU signal received at the cognitive node not only depends on the distance between the PU and the SU, but also relates to other wireless propagation characteristics, such as shadow fading, multipath, and noise. According to References [15,16], the formulation among the level of fading and the related parameters at SUs is as follows:

(1) $$PL\left(d\right)\left[\mathrm{dB}\right]=\bar{PL}\left(d\right)+X_{\sigma }=\bar{PL}\left(d_{0}\right)+10n\log\frac{d}{d_{0}}+X_{\sigma }.$$

Path loss PL(d) is a function of d, which denotes the distance between the PU and the SU. $X_{\sigma }$ is subject to Gaussian distribution with a mean value of 0 and a variance of σ, i.e., $X_{\sigma }∼N\left(0,\sigma^{2}\right)$. $d_{0}$ and n represent the reference distance and the path loss factor, respectively.

Assuming that $P_{t}$ denotes the transmission power of the primary user, when the PU is present, the power received at the i-th SU can be expressed as $P_{r}^{i}\left(d_{i}\right)=P_{t}-PL\left(d_{i}\right)$. When the PU is absent, $P_{r}^{i}\left(d_{i}\right)=W_{i}$, where $W_{i}$ follows Gaussian distribution with $N\left(\bar{PL}\left(d_{i}\right),\sigma_{0}^{2}\right)$.

Assume that SUs detect the PUs based on energy detection with a threshold λ. Hence, the probability of detection of the i-th SU can be formulated as:

(2) $$\begin{array}{l} P_{d}^{i}=P\left(P_{r}^{i}>\lambda |H_{1}\right)=P\left(X_{\sigma }<P_{t}-\bar{PL}\left(d_{i}\right)-\lambda \right)\\ =Q\left(\frac{\lambda -P_{t}+\bar{PL}\left(d_{i}\right)}{\sigma }\right) \end{array},$$

where $Q\left(z\right)=1/\sqrt{2\pi }\int_{z}^{\infty }\exp\left(-x^{2}/2\right)dx$ denotes the complementary distribution function of standard normal distribution. Similarly, the probability of a false alarm of the i-th SU can be expressed as:

(3) $$P_{f}^{i}=Q\left(\frac{\lambda -\bar{PL}\left(d_{i}\right)}{\sigma_{0}}\right).$$

Although CSS may improve the sensing performance, it also provides SSDF attackers with the opportunity to undermine the sensing performance due to its nature of openness. SSDF attackers can mislead the FC and deteriorate the CSS performance by means of reporting false sensing results. Thereby, the CSS algorithm must be robust to SSDF attacks. Although some algorithms can detect malicious nodes [11], the residual malicious nodes will still affect the spectrum sensing performance. As the fraction of malicious nodes rises in cognitive radio networks, the probability of CSS detection decreases. Meanwhile, the probability of CSS false alarm ascends. Therefore, different sensing strategies are needed under different fractions of malicious nodes.

## 3. Collaborative Spectrum Sensing in CRAHNs

In this section, the graph cut algorithm used regularly in computer vision is introduced into CSS, where the graph cut-based maximum a posteriori estimation of spectrum occupancy states is realized. In the field of computer vision, graph cut algorithms are often used to derive the minimum energy of an image [17,18], and divide this image into ‘foreground’ and ‘background’. Similarly, in CSS, all SUs nodes are expected to decide whether the PU is ‘absent’ or ‘present’ at each node.

Based on the spectrum state of each node in CRAHNs, a graph of spectrum occupancy status is constructed in this section. Each SU is regarded as a graph pixel and links $\left(v_{i},v_{j}\right)$ between neighboring nodes i and j are established based on their topology information. Hence, an undirected graph of spectrum occupancy status (GSOS) can be built. In computer vision, two terminals (source and sink) are added to the image, and each node in the image relates to two terminals [17]. Similarly, source node O, denoting spectrum occupied, and terminal node U, denoting spectrum unoccupied, and the connection between each node and two endpoints $l\left(v_{o},v_{j}\right)$, $l\left(v_{j},v_{u}\right)$ are added in the GSOS. In this way, dividing SU status in CSS is equivalent to image segmentation in computer vision, and can be further processed by existing graph cut algorithms.

### 3.1. Maximum a Posteriori Probability of CSS

Given the spectrum state of the i-th secondary user $x_{i}$, where $\left\{x_{i}=1,0\right\}$ meaning that its spectrum is occupied or vacant, respectively, the PU signal energy $y_{i}$ received at the i-th secondary user is conditionally dependent on $x_{i}$, and the conditional probability density function is $f\left\{y_{i}|x_{i}\right\}$. Therefore, considering a cognitive radio network with N SU nodes with spectrum state $x=\left\{x_{1},x_{2},\ldots ,x_{N}\right\}$, the power density function of the signal power received by each SU node is denoted as:

(4) $$p\left(y|x\right)=\prod_{i=1}^{N}f\left(y_{i}|x_{i}\right)=\prod_{i=1}^{N}f{\left(y_{i}|x_{i}=1\right)}^{x_{i}}f{\left(y_{i}|x_{i}=0\right)}^{1-x_{i}}.$$

where $y=\left\{y_{1},y_{2},\ldots ,y_{N}\right\}$. In cognitive radio networks, the prior distribution of SU nodes can be considered as a conditional Markov random field (MRF) [19], which is a commonly used model for location scenes with numerable patches having the same spectrum status. Thereby, the prior distribution of each node state p(x) can be expressed as follows [19]:

(5) $$p\left(x\right)\propto \exp\left\{\frac{1}{2}\sum_{i=1}^{N}\sum_{i=1}^{N}\beta_{ij}\left[x_{i}x_{j}+\left(1-x_{i}\right)\left(1-x_{j}\right)\right]\right\},$$

where $\beta_{ij}$ stands for the relationship between two SU nodes. When the i-th secondary user and j-th secondary user are adjacent nodes, we have $\beta_{ij}=\beta_{ji}=\beta$, where β is constant; otherwise, $\beta_{ij}=\beta_{ji}=0$.

According to the Bayes criterion, the posterior probabilities of the spectrum state of the network nodes p(x|y) can be written as:

(6) $$p\left(x|y\right)=\frac{p\left(x,y\right)}{p\left(y\right)}=\frac{p\left(x\right)p\left(y|x\right)}{p\left(y\right)}.$$

Namely, the logarithm of the posterior probability, lnp(x|y), can be formulated as:

(7) $$L\left(x\right)=\sum_{i=1}^{N}x_{i}\ln\frac{f\left(y_{i}|x_{i}=1\right)}{f\left(y_{i}|x_{i}=0\right)}+\frac{1}{2}\sum_{i=1}^{N}\sum_{j=1}^{N}\left(x_{i}x_{j}+\left(1-x_{i}\right)\left(1-x_{j}\right)\right)+K,$$

where $\frac{f\left(y_{i}|x_{i}=1\right)}{f\left(y_{i}|x_{i}=0\right)}$ represents the likelihood ratio of the SUs. The constant K, which is independent of the spectrum states, can be expressed as:

(8) $$K=\sum_{i=1}^{N}p\left(y_{i}|x_{i}=0\right)-\ln y.$$

### 3.2. Parameter Setting of the Graph Cut

The minimum energy of the graph according to the maximum flow/minimum cut algorithm [18] can be written as [20]:

(9) $$E\left(L\right)=\sum_{p\in P}D_{p}\left(L_{p}\right)+\sum_{\left(p,q\right)\in \phi }V_{p,q}\left(L_{p},L_{q}\right),$$

where $L=\left\{L_{p},p\in P\right\}$, $D_{p}\left(\cdot \right)$, $V_{p,q}\left(\cdot \right)$, and ϕ denote the value of each pixel of the penalty function for each node’s value, the interaction function between the pixels p and q, and the set of all adjacent nodes, respectively.

This paper makes some adoptions to build the connection between the results of graph cut and the maximum a posteriori estimation of the SUs’ spectrum status in CSS. Similarly with the penalty function $D_{p}\left(\cdot \right)$ and the interaction function $V_{p,q}\left(\cdot \right)$ in the graph cut, the sensing result cost functions $c\left(v_{i},v_{o}\right)$, $c\left(v_{u},v_{i}\right)$ and the topological information cost function $c\left(v_{i},v_{j}\right)$ are introduced, respectively.

1. Sensing results cost function:

(10) $$c\left(v_{i},v_{o}\right)=\ln\left(\frac{f\left(y_{i}|x_{i}=1\right)}{f\left(y_{i}|x_{i}=0\right)}\right)=\lambda_{i},$$

(11) $$c\left(v_{u},v_{i}\right)=\ln\eta ,$$

where η is set to 1, and thus $c\left(v_{u},v_{i}\right)=0$. From Equations (10) and (11), we can derive that when $c\left(v_{i},v_{o}\right)>c\left(v_{u},v_{i}\right)$, the i-th node prefers to decide that the spectrum status at the i-th node is idle, which is equal to the decision of the independent spectrum sensing.

When it comes to making a hard decision, $c^{\prime }\left(v_{i},v_{o}\right)=1$ and $c^{\prime }\left(v_{u},v_{i}\right)=0$ when the likelihood ratio function is greater than 1; otherwise, $c^{\prime }\left(v_{i},v_{o}\right)=0$ and $c^{\prime }\left(v_{u},v_{i}\right)=1$. Thus, the cognitive node reports this 1-bit hard decision data to the FC.

2. Topological information cost function:

(12) $$c\left(v_{i},v_{j}\right)=\{\begin{array}{cc} \beta , & i\;and\;j\;are\;neighbors\\ 0, & otherwise \end{array}.$$

The topological information cost function denotes the correlation of the spectrum status between two nodes. The topological information cost function $c\left(v_{i},v_{j}\right)$ is equal to β when the i-th node and j-th node are neighbors to each other topologically. It is reasonable that adjacent nodes prefer to undergo the same spectrum status and thus β>0.

Based on the aforementioned cost functions, the energy function of GSOS can be written as:

(13) $$C\left(x\right)=\sum_{i=1}^{N}x_{i}c\left(v_{i},v_{o}\right)+\sum_{i=1}^{N}\left(1-x_{i}\right)c\left(v_{u},v_{i}\right)+\frac{1}{2}\sum_{i=1}^{N}\sum_{j=1}^{N}c\left(v_{i},v_{j}\right){\left(x_{i}-x_{j}\right)}^{2},$$

which differs from the negative part of the posterior probability function −L(x) by a constant K. Suppose that minimum of C(x) is taken at $x=x_{0}$, it is obvious that $L\left(x_{0}\right)$ is the maximum of L(x). In other words, graph cut algorithms minimize the energy function of an image, C(x), and consequently derive the maximum a posteriori estimation in CSS.

## 4. Spectrum Sensing Strategy Selection Based on Reputation Value

In the field of CSS, reputation value theory is often applied to distinguish HSUs from SSDF attackers [21,22]. In this section, reputation value theory is innovatively adopted to a select sensing strategy between CSS and independent sensing.

Massive MSUs in CRAHNs will deteriorate the performance of CSS dramatically, which contradicts the belief that CSS should improve the sensing performance. Different from CSS, independent sensing ensures the independence of each node’s sensing results, which means that MSUs have no chance to interfere with HSUs in independent sensing. Therefore, it is advisable to select independent spectrum sensing when enormous malicious nodes exist, and vice versa. However, the fraction of MSUs in CRAHNs is not easy to determine.

Though the fraction of MSUs is unknown for each SU, local sensing data and CCS results can be easily obtained at an HSU without additional hardware costs. This information can be further exploited to evaluate the performance of independent spectrum sensing and CSS. At each SU node, the performance of independent sensing is compared with that of CSS. When the results of the former are the same as those of the latter, the reputation value is increased by 1; when they are different, the reputation value is reduced by 1. Therefore, collaborative sensing results are trustworthy when the reputation value is high. Likewise, independent sensing decisions are reliable when the reputation value is low. Thereby, a specific reputation threshold should be investigated. CSS results are selected when the reputation value exceeds the reputation threshold, and independent sensing results are chosen when the reputation value is below the threshold.

To elaborate upon the sensing strategies selection, the reputation value is analyzed at an honest node. Assuming that the sensing result at moment t is denoted as $u_{t}$ with detection probability $P_{d}^{I}$ and false alarm probability $P_{f}^{I}$, $g_{t}$ represents the CSS result at moment t with $P_{d}^{C}$ and $P_{f}^{C}$ representing the detection probability and false alarm probability, respectively. Let $e_{t}$ denote the comparison between the sensing result of the FC and the sensing result of the cognitive node.

It is assumed that the result of independent spectrum sensing is equals to that of CSS at moment t, denoted as:

(14) $$e_{t}=\{\begin{array}{c} 1,u_{t}=g_{t}\\ 0,u_{t}\neq g_{t} \end{array}.$$

$P\left\{e_{t}=1\right\}=p$ represents the probability that the CSS result will be identical to the independent spectrum sensing result, and it can be written as follows:

(15) $$\begin{array}{ll} p & =\sum_{j\in \left\{0,1\right\}}P\left\{Q_{j}\right\}\left(P\left\{g_{t}=1,u_{t}=1|Q_{j}\right\}+P\left\{g_{t}=0,u_{t}=0|Q_{j}\right\}\right)\\ & =\sum_{j\in \left\{0,1\right\}}P\left\{Q_{j}\right\}\left(P\left\{g_{t}=1|Q_{j}\right\}\cdot P\left\{u_{t}=1|Q_{j}\right\}+P\left\{g_{t}=0|Q_{j}\right\}\cdot P\left\{u_{t}=0|Q_{j}\right\}\right)\\ & =P\left\{Q_{1}\right\}\left(P_{d}^{I}P_{d}^{C}+\left(1-P_{d}^{I}\right)\left(1-P_{d}^{C}\right)\right)+P\left\{Q_{0}\right\}\left(P_{f}^{I}P_{f}^{C}+\left(1-P_{f}^{I}\right)\left(1-P_{f}^{C}\right)\right)\\ & =1-P\left\{Q_{1}\right\}\left(P_{d}^{I}+P_{d}^{C}-2P_{d}^{I}P_{d}^{C}\right)-P\left\{Q_{0}\right\}\left(P_{f}^{I}+P_{f}^{C}-2P_{f}^{I}P_{f}^{C}\right) \end{array},$$

$P\left\{e_{t}=0\right\}=q$ denotes the probability that the CSS result will be different from the independent spectrum sensing result, and it can be formulated as follows:

(16) $$\begin{array}{ll} q & =1-p\\ & =P\left\{Q_{1}\right\}\left(P_{d}^{I}+P_{d}^{C}-2P_{d}^{I}P_{d}^{C}\right)+P\left\{Q_{0}\right\}\left(P_{f}^{I}+P_{f}^{C}-2P_{f}^{I}P_{f}^{C}\right) \end{array},$$

The partial derivative of p on $P_{d}^{C}$ and $P_{f}^{C}$ can be expressed as:

(17) $$\frac{\partial p}{\partial P_{d}^{C}}=-P\left\{Q_{1}^{A}\right\}\left(1-2P_{d}^{I}\right),$$

(18) $$\frac{\partial p}{\partial P_{f}^{C}}=-P\left\{Q_{0}\right\}\left(1-2P_{f}^{I}\right),$$

where it is reasonably assumed that $1>P_{d}^{I}>\frac{1}{2}>P_{f}^{I}>1$ and $1>P_{d}^{C}>\frac{1}{2}>P_{f}^{C}>1$. Thus, $\frac{\partial p}{\partial P_{d}^{C}}>0$ and $\frac{\partial p}{\partial P_{f}^{C}}<0$ hold true. p decreases with the increase of $P_{d}^{C}$ at interval [0.5,1]. Likewise, p increases with the increase of $P_{f}^{C}$ at interval [0.5,1]. When $\left(P_{d}^{C},P_{f}^{C}\right)=\left(0.5,0.5\right)$, p achieves the minimum $P_{\min}$, which can be denoted as:

(19) $$P_{\min}=1-0.5\left(P\left\{Q_{1}\right\}+P\left\{Q_{0}\right\}\right)=0.5,$$

When $\left(P_{d}^{C},P_{f}^{C}\right)=\left(P_{d}^{A},P_{f}^{A}\right)$, $P_{thr}$ is derived as:

(20) $$P_{thr}=1-P\left\{Q_{1}\right\}2P_{d}^{I}\left(1-P_{d}^{I}\right)-P\left\{Q_{0}\right\}2P_{f}^{I}\left(1-P_{f}^{I}\right),$$

When $\left(P_{d}^{C},P_{f}^{C}\right)=\left(1,0\right)$, the maximum of p is formulated as:

(21) $$\begin{array}{ll} P_{\max} & =1-P\left\{Q_{1}\right\}\left(P_{d}^{I}+1-2P_{d}^{I}\right)-P\left\{Q_{0}\right\}P_{f}^{I}\\ & =1-P\left\{Q_{1}\right\}\left(1-P_{d}^{I}\right)-P\left\{Q_{0}\right\}P_{f}^{I} \end{array},$$

Obviously, $p\geq P_{Thr}$ is obtained when $P_{d}^{C}\geq P_{d}^{I}$, $P_{f}^{C}\leq P_{f}^{I}$; $p<P_{Thr}$ is obtained when $P_{d}^{C}<P_{d}^{I}$, $P_{f}^{C}>P_{f}^{I}$. Therefore, p can be used as a performance metric to compare the performance of CSS and that of independent spectrum sensing. When $p\geq P_{Thr}$, CSS outperforms independent sensing; when $p<P_{Thr}$, independent spectrum sensing outperforms CSS. To achieve better sensing performance, the CSS results rather than the independent sensing results are chosen as the final decision when $p\geq P_{Thr}$; meanwhile, the independent spectrum sensing results are selected when $p<P_{Thr}$. The abovementioned procedure is termed the spectrum sensing strategies selection (4S) algorithm.

## 5. Selection of Reputation Value Threshold

For the purpose of obtaining optimal sensing results, the 4S algorithm is expected to select the CSS results when the results of CSS are excellent; the independent spectrum sensing decision is deemed as the final decision in the 4S algorithm when independent sensing outperforms CSS. The first type of sensing error $P_{e1}$ and the second type of sensing error $P_{e2}$ are defined as the choice of the independent spectrum sensing results when the CSS decisions are optimal and should be selected, and the selection of the CSS results when the performance of the independent sensing is better than that of CSS, respectively. To reduce the sum of the first type of strategy selection error and second type of strategy selection error for the i-th SU, i.e., $P_{e}^{i}=P_{e1}+P_{e2}$, an optimal reputation value threshold can be designed as follows.

**Definition** **1.** *The reputation value of the i-th SU—E is recorded by comparing the sensing results of the FC with the local sensing results, i.e., $e_{t}$ over a limited time window from $t_{0}$ to $t_{0}+T$.*

(22) $$E=\sum_{t=t_{0}}^{t_{0}+T}e_{t},$$

*where*$e_{t}$*follows the Bernoulli distribution with the parameter*ρ*. Therefore,*E*is subject to the binomial distribution. According to Reference [23], when*T*,*$P\left\{e_{t}=1\right\}=p$*, and*$P\left\{e_{t}=0\right\}=q$*satisfy the following condition,*

(23) $$\left|\frac{1}{\sqrt{T}}\left(\sqrt{\frac{q}{p}}-\sqrt{\frac{p}{q}}\right)\right|<0.3,$$

E*approximately follows a normal distribution, i.e.,*E∼N(Tp,Tpq)*. The probability density functions of*E*can be formulated as:*

(24) $$f\left(E\right)=\frac{1}{\sqrt{2\pi Tpq}}\exp\left(-\frac{{\left(E-Tp\right)}^{2}}{2Tpq}\right).$$

*Considering that*p≫q*, it’s reasonable to assume that*$\sqrt{q/p}\approx 0$. *Thus, the formula (23) can be simplified as*

(25) $$T>\frac{p}{0.09q}\approx 11\frac{p}{q},$$

*Under the condition from Equation (25), an appropriate reputation value threshold can be derived to minimize the probability of total strategy selection error.*

For the i-th SU, when $p>P_{Thr}$, the CSS results are optimal, and the detection probability of CSS the false alarm probability of independent sensing are greater than the detection probability of independent sensing and the false alarm probability of CSS, respectively, i.e., $P_{d}^{C}>P_{d}^{I}$ and $P_{f}^{C}<P_{f}^{I}$; conversely, the independent spectrum sensing performance is better and $P_{d}^{C}<P_{d}^{I}$, $P_{f}^{C}>P_{f}^{I}$. A proper reputation value threshold is η investigated for E, and the cooperative spectrum sensing results are chosen when E>η; conversely, the independent spectrum sensing results should be selected when E<η. Thus another definition of the first type of sensing error can be expressed as the probability of E>η when p is less than a given threshold $P_{Thr}$, i.e., $P\left\{E>\eta |p<P_{Thr}\right\}$; the second type of sensing error can be denoted as the probability of E<η when $p>P_{Thr}$, i.e., $P\left\{E<\eta |p>P_{Thr}\right\}$.

The probability of the first type of sensing error, $P\left\{E>\eta |p<P_{Thr}\right\}$ can be formulated as:

(26) $$P\left\{E>\eta |p<P_{Thr}\right\}=\int_{\eta }^{+\infty }f\left(E\right)dE=\int_{\eta }^{+\infty }\frac{1}{\sqrt{2\pi Tpq}}\exp\left(-\frac{{\left(E-Tp\right)}^{2}}{2Tpq}\right)dE,$$

The probability of the second type of sensing error, $P\left\{E<\eta |p>P_{Thr}\right\}$ can be formed as:

(27) $$P\left\{E<\eta |p>P_{Thr}\right\}=\int_{0}^{\eta }f\left(E\right)dE=\int_{0}^{\eta }\frac{1}{\sqrt{2\pi Tp\left(1-p\right)}}\exp\left(-\frac{{\left(E-Tp\right)}^{2}}{2Tp\left(1-p\right)}\right)dE.$$

Let us assume that the cognitive radio network has a massive amount of honest cognitive nodes, thus ${\left(P_{d}^{C}\right)}_{\max}\approx 1$ and ${\left(P_{f}^{C}\right)}_{\min}\approx 0$ according to Reference [24]. In accordance with Reference [16], under the best SSDF attack, the performance of the cognitive radio network is at its worst with ${\left(P_{d}^{C}\right)}_{\min}=0.5$ and ${\left(P_{f}^{C}\right)}_{\max}=0.5$.

Given that θ(p) represents the probability density function of $P\left\{e_{t}=1\right\}=p$, it is assumed that p is subject to uniform distribution at $\left[P_{\min},P_{\max}\right]$ under the attack of malicious nodes; that is:

(28) $$\theta \left(p\right)=\{\begin{array}{cc} \frac{1}{P_{\max}-P_{\min}}, & P_{\min}\leq p\leq P_{\max}\\ 0, & others \end{array}.$$

Therefore, the total strategy selection error probability for the i-th SU $P_{e}^{i}$ can be written as:

(29) $$\begin{array}{ll} P_{e}^{i} & =\int_{0.5}^{P_{thr}}\theta \left(p\right)\cdot \int_{\eta }^{+\infty }f\left(E\right)dEd\theta +\int_{P_{thr}}^{1}p\left(\theta \right)\cdot \int_{0}^{\eta }f\left(E\right)dEdp\\ & =\int_{0.5}^{P_{thr}}\theta \left(p\right)\cdot \int_{\eta }^{+\infty }f\left(\frac{1}{\sqrt{2\pi Tp\left(1-p\right)}}\exp\left(-\frac{{\left(E-Tp\right)}^{2}}{2Tp\left(1-p\right)}\right)\right)dEdp\\ & +\int_{P_{thr}}^{1}\theta \left(p\right)\cdot \int_{0}^{\eta }f\left(\frac{1}{\sqrt{2\pi Tp\left(1-p\right)}}\exp\left(-\frac{{\left(E-Tp\right)}^{2}}{2Tp\left(1-p\right)}\right)\right)dEdp \end{array},$$

The partial derivative of the total strategy selection error probability over the reputation value threshold η can be computed as:

(30) $$\begin{array}{ll} \frac{\partial P_{e}^{i}}{\partial \eta } & =\frac{\partial \left(\int_{P_{\min}}^{P_{thr}}\theta \left(p\right)\cdot \int_{\eta }^{+\infty }f\left(E\right)dEdp+\int_{P_{thr}}^{P_{\max}}\theta \left(p\right)\cdot \int_{0}^{\eta }f\left(E\right)dEdp\right)}{\partial \eta }\\ & =-\int_{P_{\min}}^{P_{thr}}\theta \left(p\right)\cdot f\left(\eta \right)dp+\int_{P_{thr}}^{P_{\max}}\theta \left(p\right)\cdot f\left(\eta \right)dp\\ & =\frac{1}{P_{\max}-P_{\min}}(\int_{P_{\min}}^{P_{thr}}-\frac{1}{\sqrt{2\pi Tp\left(1-p\right)}}\exp\left(-\frac{{\left(\eta -Tp\right)}^{2}}{2Tp\left(1-p\right)}\right)dp\\ & +\int_{P_{thr}}^{P_{\max}}\frac{1}{\sqrt{2\pi Tp\left(1-p\right)}}\exp\left(-\frac{{\left(\eta -Tp\right)}^{2}}{2Tp\left(1-p\right)}\right)dp) \end{array}.$$

**Proposition** **1.** *The solution for the optimal reputation value exists.*

**Proof.**  See Appendix A. □

According to Proposition 1, the solution of $\frac{\partial P_{e}^{i}}{\partial \eta }=0$ exists, and $\frac{\partial P_{e}^{i}}{\partial \eta }=0$ can be expressed as:

(31) $$\begin{array}{l} \frac{1}{P_{\max}-P_{\min}}(\int_{P_{\min}}^{P_{thr}}-\frac{1}{\sqrt{2\pi Tp\left(1-p\right)}}\exp\left(-\frac{{\left(\eta -Tp\right)}^{2}}{2Tp\left(1-p\right)}\right)dp\\ +\int_{P_{thr}}^{P_{\max}}\frac{1}{\sqrt{2\pi Tp\left(1-p\right)}}\exp\left(-\frac{{\left(\eta -Tp\right)}^{2}}{2Tp\left(1-p\right)}\right)dp)=0 \end{array}.$$

The solution of the above equation is the optimal reputation threshold of the 4S algorithm. To reduce the computational complexity, an error function is applied to approximate the formula $\frac{\partial P_{e}^{i}}{\partial \eta }=0$ as follows (see Appendix B):

(32) $$\frac{\partial P_{e}^{i}}{\partial \eta }=\frac{1}{2\left(P_{\max}-P_{\min}\right)}\left(\mathrm{erf}\left(\frac{P_{\max}}{\sqrt{2T\eta \left(1-\eta \right)}}\right)+\mathrm{erf}\left(\frac{P_{\min}}{\sqrt{2T\eta \left(1-\eta \right)}}\right)-2\mathrm{erf}\left(\frac{P_{\mathrm{thr}}}{\sqrt{2T\eta \left(1-\eta \right)}}\right)\right),$$

where erf(x) is the error function. We define optimal threshold function h(η) as:

(33) $$h\left(\eta \right)=\mathrm{erf}\left(\frac{P_{\max}}{\sqrt{2T\eta \left(1-\eta \right)}}\right)+\mathrm{erf}\left(\frac{P_{\min}}{\sqrt{2T\eta \left(1-\eta \right)}}\right)-2\mathrm{erf}\left(\frac{P_{\mathrm{thr}}}{\sqrt{2T\eta \left(1-\eta \right)}}\right).$$

It can be seen that h(η) is equal to zero when $\eta =\eta_{0}$, $\frac{\partial P_{e}^{i}}{\partial \eta }<0$ when $\eta <\eta_{0}$, and $\frac{\partial P_{e}^{i}}{\partial \eta }>0$ when $\eta >\eta_{0}$. Namely, the total strategy selection error probability monotonically decreases at interval $\left(0.5,\eta_{0}\right)$ and ascends at interval $\left(\eta_{0},1\right)$. Accordingly, the total strategy selection error probability for the i-th SU reaches its minimum at $\eta =\eta_{0}$.

## 6. Performance Analysis

This section analyzes and investigates the performances of independent spectrum sensing, CSS, and the proposed 4S algorithm in CRAHNs under various SSDF attack strategies.

### 6.1. Parameter Setting

In this section, a centralized cognitive radio network is considered, where three sensing algorithms are applied and simulated. The PU is assumed to be located at the center of the cognitive radio network, and accesses the licensed band with a probability of 0.5. The power and the bandwidth of the PU emitted to the cognitive radio network reaches 20 watts and 900 MHz, respectively. All of the SUs’ locations, including HSUs and MSUs, are subject to a Poisson point process with a density of $8\times 10^{-5}/m^{2}$. Wireless channel propagation is modeled as Rayleigh distribution with mean of 1, and the path loss factor is 4. Background noise n(t) follows normal distribution with $N\left(0,10^{-14}\right)$. Prior information about partial spectrum homogeneity, β, is equal to 0.4.

### 6.2. SSDF Attack Strategies

At first, spectrum sensing is implemented at each node. Afterwards, SSDF attackers resolve their report data based on their sensing results and attacking strategies. SSDF attacking strategies can be divided into four ways [25], listed as follows.

1. “Always occupied” attack: To access spectrum resources alone, MSUs send reports of being occupied by the PU to force the FC to regard the spectrum status as always occupied. Thus, SUs have no privilege to access the spectrum band. Therefore, the total spectrum utilization efficiency is reduced.

2. “Always idle” attack: When the spectrum status is occupied, malicious nodes send ‘idle’ reports to deliberately mislead the FC to allocate spectrum resources to SUs. As a result, the quality of communication is worsened.

3. “Always false” attack: Sensing data reported to the FC by MSUs is always completely contrary to the sensing results. That is, when the malicious user detects the existence of the PU, the malicious user sends an ‘idle’ report to the FC; if the malicious user finds that the spectrum status is idle, the malicious user sends an ‘occupied’ report to the FC.

4. Possible attack: To avoid being detected and eliminated by the FC, the malicious user reports not only false results but also true results under a specific probability.

### 6.3. Simulation Results

Considering the goal of minimizing the average total strategy selection error probability for all HSUs, $\bar{P_{e}}$, we employ the probability of correctness for HSUs as a performance metric, which is given by:

(34) $$P_{c}=1-\bar{P_{e}}=1-\frac{\sum_{i=1}^{N-M}\kappa^{i}Pb_{C}^{i}+\left(1-\kappa^{i}\right)Pb_{I}^{i}}{N-M},$$

where $Pb_{C}^{i}$ and $Pb_{I}^{i}$ denote the sensing error of CSS and the sensing error of independent sensing, respectively; $\kappa^{i}=0$ and $\kappa^{i}=1$ indicate that the CSS results are selected and that the independent sensing results are selected for i-th SU, respectively.

Assuming that the sensing method with better performance is always chosen in the 4S algorithm, we can derive the upper bound of the probability of correctness, $\overset{⌢}{P_{c}}$, which is given by:

(35) $$\overset{⌢}{P_{c}}=1-\frac{\sum_{i=1}^{N-M}\min\left\{Pb_{C}^{i},Pb_{I}^{i}\right\}}{N}.$$

When the number of SUs, N, is constant, the fraction of SSDF attackers is denoted as:

(36) $$\alpha =\frac{\mathrm{number}\;\mathrm{of}\;\mathrm{SSDF}\;\mathrm{attackers}}{\mathrm{number}\;\mathrm{of}\;\mathrm{honest}\;\mathrm{SUs}\;+\;\mathrm{number}\;\mathrm{of}\;\mathrm{SSDF}\;\mathrm{attackers}\;}.$$

**Figure 2:**
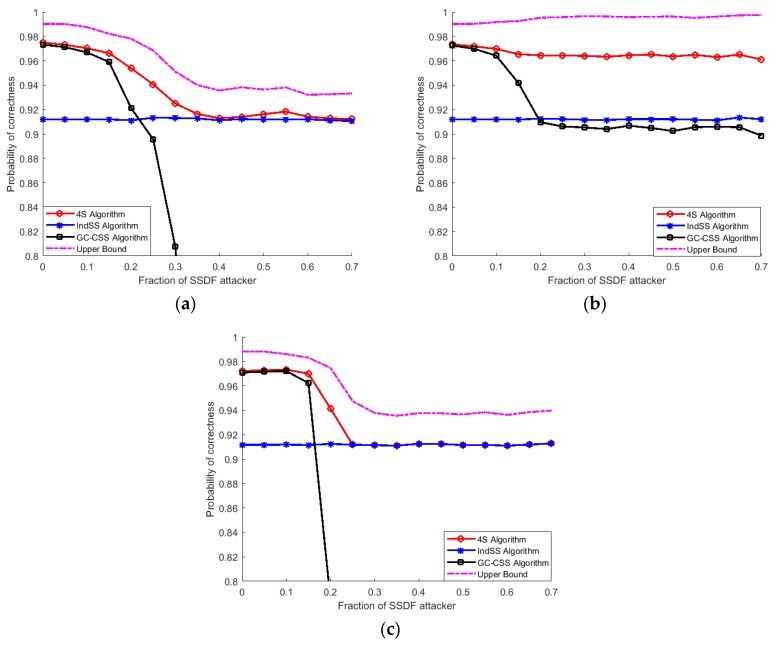
Comparison of three sensing strategies under various attack strategies. (**a**) “Always false” attack; (**b**) “always idle” attack; (**c**) “always occupied” attack.

Figure 2 shows that independent spectrum sensing is not affected by the fraction of MSUs at all and that CSS is sensitive to the fraction of MSUs and performs worse when it comes to a high fraction of MSUs. Moreover, ‘always occupied’ attacks have greatest impacts on CSS and ‘always idle’ attacks have the lowest effects on CSS. This is mainly due to the large coverage area of CRAHNs, which means that most cognitive nodes are out of the PU’s coverage and may fall into an ‘idle’ status. MSUs out of the PU’s coverage, which adopt an ‘always idle’ strategy, report errorless data to the FC, and vice versa. In Figure 2a, when the fraction of SSDF attackers is high, the performance of CSS is low and thus independent sensing is selected as the final decision of the 4S algorithm. Therefore, the performance of the 4S algorithm converges with the performance of independent sensing. In Figure 2b, the flattening of the plots is caused because SUs out of the PU’s coverage report errorless data to the FC. The convergence value depends on the ratio of the number of SUs out of the PU’s coverage to the number of SUs in the PU’s coverage.

It also can be seen from Figure 2 that there is a gap between the performance of the 4S algorithm and the upper bound of the 4S algorithm. Firstly, we assume that ${\left(P_{d}^{C}\right)}_{\max}\approx 1$ and ${\left(P_{f}^{C}\right)}_{\min}\approx 0$; when these are not perfectly satisfied, the threshold selection is not optimal. Secondly, in the above discussion, the total strategy selection error $P_{e}^{i}$ reaches a minimum value, which is equal to 0. This means that there are still some cases in which SUs make the wrong choice in strategy selection. Therefore, the upper bound of the 4S algorithm cannot be reached.

Overall, Figure 2 proves that the 4S algorithm performs much better than CSS when the fraction of MSUs is high and slightly worse than CSS in the case of a limited fraction of MSUs. Moreover, the 4S algorithm is almost always superior to independent spectrum sensing, except in the case of moderate to high fractions of MSUs under an “always occupied” attack.

**Figure 3:**
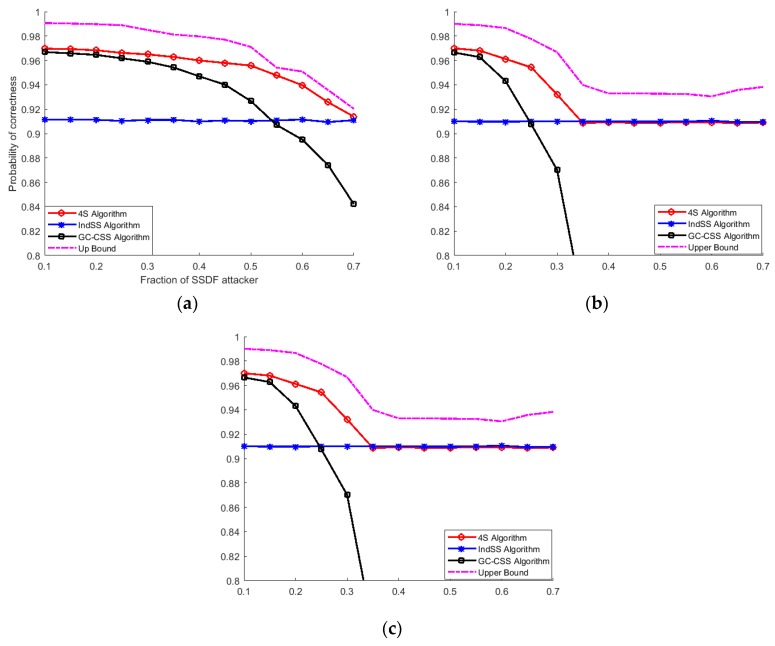
Comparison of three sensing strategies under possible attack with a hard decision. (**a**) Possible attack with probability = 0.2; (**b**) possible attack with probability = 0.5; (**c**) possible attack with probability = 0.8.

**Figure 4:**
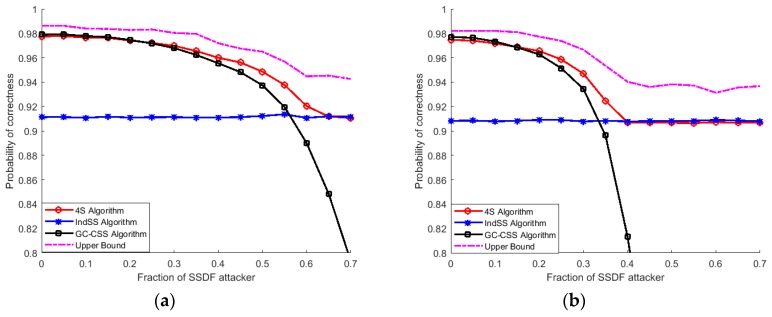

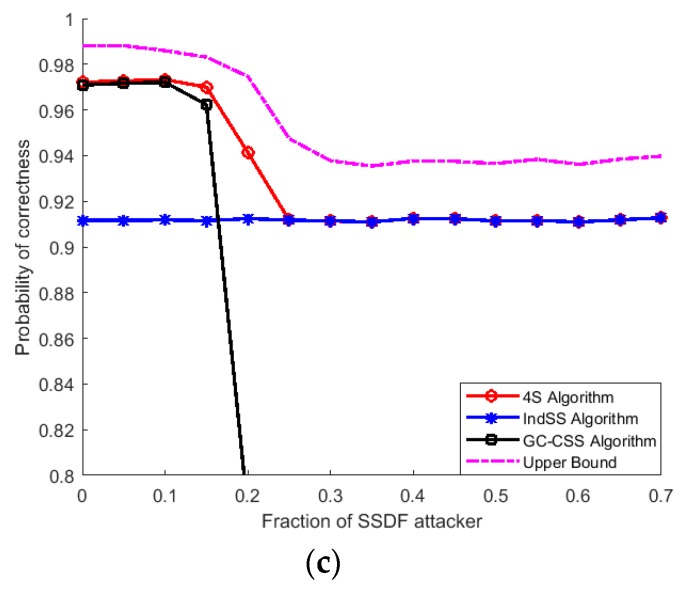
Comparison of three sensing strategies under possible attack with a soft decision. (**a**) possible attack with probability = 0.2; (**b**) possible attack with probability = 0.5; (**c**) possible attack with probability = 0.8.

Figure 3 and Figure 4 show the influence of probabilistic attacks on the performance of three sensing algorithms with a hard decision and with a soft decision [4], respectively. It was demonstrated that CSS with a soft decision is vulnerable to malicious attacks and the performance of CSS with a soft decision is worse than that of CSS with a hard decision. This is mainly due to the robustness of the hard decision topological information cost function. Compared with the soft decision topological information function, the hard one causes information losses to MSUs, which results in the poor performance of MSUs. Therefore, CSS with a hard decision perform better than CSS with a soft decision under a high fraction of MSUs. Although the performance of the 4S algorithm decays as a result of the decline of the CSS performance as the fraction of MSUs rises, the former still outperforms CSS and independent spectrum sensing. As the probability of attack increases, the curve of the 4S algorithm merges with that of independent sensing, which means that the 4S algorithm selects the independent sensing results when the CSS results are deeply affected by malicious nodes.

Owing to spectrum heterogeneity in CRAHNs and various fractions of MSUs, the performance of CSS and independent sensing among cognitive nodes differs. In the 4S algorithm, when the fraction of MSUs is relatively low in networks, the majority of SUs tend to select the CSS results as the final decision; thus, the performance of the 4S algorithm is roughly the same as that of CSS. When the number of MSUs increases, the performance of CSS gets worse. Therefore, it is reasonable for the 4S algorithm to select the insensitive independent sensing results as the final decision rather than the CSS results. To sum up, the 4S algorithm shows robustness under SSDF attack.

## 7. Conclusions

In this paper, we developed an adaptive deciding mechanism against SSDF attacks. Independent sensing and CSS have different sensitivities to the fraction of malicious nodes. We proposed the 4S algorithm, in which spectrum sensing is designed to adaptively select the better results between CSS and independent sensing under various attacking fractions of MSUs. The algorithm evaluates the performance of CSS and independent sensing through comparing reputation values. The simulation results clearly verified the effectiveness of the proposed 4S algorithm under varying malicious node attack strategies. The performance of the 4S algorithm is comparable ti that of CSS when the fraction of MSUs is high and it outperforms CSS when there is a low fraction of malicious nodes.

## Appendix A

By substituting $p=\frac{P_{\max}-P_{\mathrm{thr}}}{P_{\mathrm{thr}}-P_{\min}}\left(\omega -P_{\min}\right)$ in $\int_{P_{\mathrm{thr}}}^{P_{\max}}\frac{1}{\sqrt{2\pi Tp\left(1-p\right)}}\exp\left(-\frac{{\left(\eta -Tp\right)}^{2}}{2Tp\left(1-p\right)}\right)dp$, we obtain

(A1) $$\int_{P_{\min}}^{P_{\mathrm{thr}}}\frac{1}{\sqrt{2\pi Tp\left(1-p\right)}}\exp\left(-\frac{{\left(\eta -Tp\right)}^{2}}{2Tp\left(1-p\right)}\right)\cdot \frac{P_{\max}-P_{\mathrm{thr}}}{P_{\mathrm{thr}}-P_{\min}}d\omega .$$

To prove that solution of $\frac{\partial P_{e}^{i}}{\partial \eta }=0$ exists, we first prove that $\frac{\partial P_{e}^{i}}{\partial \eta }<0$ when $\eta =P_{\min}$ and $\frac{\partial P_{e}^{i}}{\partial \eta }>0$ when $\eta =P_{\max}$. Then $\frac{\partial P_{e}^{i}}{\partial \eta }{|}_{\eta =P_{\max}}\geq 0$ needs to be proved. Accordingly, we obtain

(A2) $$\int_{P_{\min}}^{P_{\mathrm{thr}}}\frac{1}{\sqrt{2\pi Tp\left(1-p\right)}}\exp\left(-\frac{{\left(\eta -Tp\right)}^{2}}{2Tp\left(1-p\right)}\right)\cdot \frac{P_{\max}-P_{\mathrm{thr}}}{P_{\mathrm{thr}}-P_{\min}}d\omega \geq \int_{P_{\min}}^{P_{\mathrm{thr}}}\frac{1}{\sqrt{2\pi Tp\left(1-p\right)}}\exp\left(-\frac{{\left(\eta -Tp\right)}^{2}}{2Tp\left(1-p\right)}\right)dp.$$

Thus, the above inequation is proven if the following inequation is proved to be true:

(A3) $$\frac{1}{\sqrt{2\pi T\theta \left(1-\theta \right)}}\exp\left(-\frac{{\left(\eta -Tp\right)}^{2}}{2Tp\left(1-p\right)}\right)\cdot \frac{P_{\max}-P_{\mathrm{thr}}}{P_{\mathrm{thr}}-P_{\min}}\geq \frac{1}{\sqrt{2\pi T\omega \left(1-\omega \right)}}\exp\left(-\frac{{\left(\eta -T\omega \right)}^{2}}{2T\omega \left(1-\omega \right)}\right),$$

where $p=\frac{P_{\max}-P_{\mathrm{thr}}}{P_{\mathrm{thr}}-P_{\min}}\left(\omega -P_{\min}\right)$.

That is,

(A4) $$\exp\left(\frac{{\left(\eta -T\omega \right)}^{2}}{2T\omega \left(1-\omega \right)}-\frac{{\left(\eta -Tp\right)}^{2}}{2Tp\left(1-p\right)}\right)\geq \frac{\sqrt{2\pi Tp\left(1-p\right)}}{\sqrt{2\pi T\omega \left(1-\omega \right)}}\cdot \frac{P_{\mathrm{thr}}-P_{\min}}{P_{\max}-P_{\mathrm{thr}}}.$$

From the above equation, we can obtain:

(A5) $$T\geq \frac{1}{\frac{{\left(\eta /T-\omega \right)}^{2}}{2\omega \left(1-\omega \right)}-\frac{{\left(\eta /T-p\right)}^{2}}{2p\left(1-p\right)}}\log\left(\frac{\sqrt{2\pi Tp\left(1-p\right)}}{\sqrt{2\pi T\omega \left(1-\omega \right)}}\cdot \frac{P_{\mathrm{thr}}-P_{\min}}{P_{\max}-P_{\mathrm{thr}}}\right).$$

It is assumed that T is large enough, thus $\frac{\partial P_{e}^{i}}{\partial \eta }{|}_{\eta =P_{\max}}\geq 0$.

Similarly, $\frac{\partial P_{e}^{i}}{\partial \eta }\leq 0$ when $\eta =P_{\min}$ can be proved.

## Appendix B

It is difficult to solve Equation (31), hence we derive an approximation of Equation (31) with a sufficiently large T.

At first, f(x) and g(x) are defined as follows:

(A6) $$f\left(x\right)=\frac{1}{\sqrt{2\pi Tx\left(1-x\right)}}\exp\left(-\frac{{\left(\eta -Tx\right)}^{2}}{2Tx\left(1-x\right)}\right),$$

(A7) g(x)=x(1−x).

At interval [η−Δ,η+Δ], we have g(η+Δ)−g(η−Δ)=2Δ(1−2η). Therefore, g(x) can be regarded as a constant g(η) at interval [η−Δ,η+Δ] if we ignore 2Δ(1−2η). Then we obtain:

(A8) $$\begin{array}{ll} \frac{\partial P_{e}^{i}}{\partial \eta } & =\int_{P_{\mathrm{thr}}}^{P_{\max}}\frac{1}{\sqrt{2\pi Tp\left(1-p\right)}}\exp\left(-\frac{{\left(\eta -Tp\right)}^{2}}{2Tp\left(1-p\right)}\right)dp-\int_{P_{\min}}^{P_{\mathrm{thr}}}\frac{1}{\sqrt{2\pi Tp\left(1-p\right)}}\exp\left(-\frac{{\left(\eta -Tp\right)}^{2}}{2Tp\left(1-p\right)}\right)dp\\ & =\int_{\Omega_{1}}\frac{1}{\sqrt{2\pi Tg\left(\eta \right)}}\exp\left(-\frac{{\left(\eta -Tp\right)}^{2}}{2Tg\left(\eta \right)}\right)dp+\varepsilon_{1}-\int_{\Omega_{2}}\frac{1}{\sqrt{2\pi Tg\left(\eta \right)}}\exp\left(-\frac{{\left(\eta -Tp\right)}^{2}}{2Tg\left(\eta \right)}\right)dp-\varepsilon_{2} \end{array},$$

where $\varepsilon_{1}$ and $\varepsilon_{2}$ are given by:

(A9) $$\left\{ \begin{array}{l} \varepsilon_{1}=\int_{\Omega_{3}}\frac{1}{\sqrt{2\pi Tg\left(p\right)}}\exp\left(-\frac{{\left(\eta -Tp\right)}^{2}}{2Tg\left(p\right)}\right)dp\\ \varepsilon_{2}=\int_{\Omega_{4}}\frac{1}{\sqrt{2\pi Tg\left(p\right)}}\exp\left(-\frac{{\left(\eta -Tp\right)}^{2}}{2Tg\left(p\right)}\right)dp \end{array}\right.,$$

and

(A10) $$\left\{ \begin{array}{l} \Omega_{1}=\left[P_{\mathrm{thr}},P_{\max}\right]\cap \left[\eta -\Delta ,\eta +\Delta \right]\\ \Omega_{2}=\left[P_{\min},P_{\mathrm{thr}}\right]\cap \left[\eta -\Delta ,\eta +\Delta \right]\\ \Omega_{3}=\left[P_{\mathrm{thr}},P_{\max}\right]-\left[\eta -\Delta ,\eta +\Delta \right]\\ \Omega_{4}=\left[P_{\min},P_{\mathrm{thr}}\right]-\left[\eta -\Delta ,\eta +\Delta \right] \end{array}\right..$$

Consider that ξ is sufficiently small, this satisfies $\max\left\{f\left(\theta \right),\theta \in \Omega_{3}\cup \Omega_{4}\right\}\leq \xi$, i.e.,

(A11) $$\frac{1}{\sqrt{2\pi Tg\left(p\right)}}\exp\left(-\frac{{\left(\eta -Tp\right)}^{2}}{2Tg\left(p\right)}\right)\leq \xi .$$

Noting that T is sufficiently large, Equation (A11) holds, thus we obtain:

(A12) max{f(η−Δ),f(η+Δ)}≤ξ.

Namely,

(A13) $$\varepsilon_{1}=\int_{\Omega_{3}}\frac{1}{\sqrt{g\left(p\right)}}\exp\left(-\frac{{\left(\eta -Tp\right)}^{2}}{2g\left(p\right)/T}\right)dp\leq \int_{\Omega_{3}}\xi dp.$$

When ξ is sufficiently small, $\varepsilon_{1}$ can be neglected. Similarly, $\varepsilon_{1}$ is sufficiently small and is neglected. Thus, we obtain:

(A14) $$\begin{array}{ll} \frac{\partial P_{e}^{i}}{\partial \eta } & \approx \int_{\Omega_{1}}\frac{1}{\sqrt{2\pi Tg\left(\eta \right)}}\exp\left(-\frac{{\left(\eta -Tp\right)}^{2}}{2Tg\left(\eta \right)}\right)dp+\zeta_{1}\\ & -\int_{\Omega_{2}}\frac{1}{\sqrt{2\pi Tg\left(\eta \right)}}\exp\left(-\frac{{\left(\eta -\theta \right)}^{2}}{2Tg\left(\eta \right)}\right)dp-\zeta_{2}\\ & =\int_{P_{\mathrm{thr}}}^{P_{\max}}\frac{1}{\sqrt{2\pi Tg\left(\eta \right)}}\exp\left(-\frac{{\left(\eta -\theta \right)}^{2}}{2Tg\left(\eta \right)}\right)dp-\int_{P_{\min}}^{P_{\mathrm{thr}}}\frac{1}{\sqrt{2Tg\left(\eta \right)}}\exp\left(-\frac{{\left(\eta -\theta \right)}^{2}}{2Tg\left(\eta \right)}\right)dp\\ & =\frac{1}{2\left(P_{\max}-P_{\min}\right)}\left(erf\left(\frac{P_{\max}}{\sqrt{2Tg\left(\eta \right)}}\right)+erf\left(\frac{P_{\min}}{\sqrt{2Tg\left(\eta \right)}}\right)-2erf\left(\frac{P_{\mathrm{thr}}}{\sqrt{2Tg\left(\eta \right)}}\right)\right) \end{array}.$$
